# Mr. Harrold, on Fractures of the Thigh

**Published:** 1806-01

**Authors:** E. Harrold

**Affiliations:** Cheshunt, Herts


### *To the Editors of the Medical and Physical Journal.*

GENTLEMEN,

I Have long thought the common method of managing fractures of the thigh extremely defective; and most surgeons, I am disposed to believe, consider those cases singularly fortunate which terminate without either lameness or deformity. The general result of this method must, I presume, have attracted the attention of most of your readers; from the considerable number (principally indeed of the lower class) who after this accident are lame, in consequence of the limb being more or less *shortened* and the *foot* being *turned out.* The *greatest* care on the part of the surgeon will, too frequently, not be followed by happier consequences; and where, from distance (more especially in country practice) and various other impediments, such constant and exact attention cannot be paid, there can be no great difficulty in guessing at the result. Compleat success in such cases is indeed so little expected in general, that unless the deformity and lameness are very great, they seldom reflect any disgrace upon the surgeon.

We are certainly very much indebted to Mr. Pott for his very judicious remarks on fractures and dislocations. I suppose no one will dispute the principle on which his practice was founded; which principle, I believe, has been the general guide to practitioners ever since, viz. to place the limb in such a position as shall relax the whole set of muscles belonging to, or in connection with, the broken bone. This principle still holds good in fractures of the thigh; but in his mode of applying it, it is by no means constantly attended with compleat success. The patient is laid upon the fractured limb, with the thigh and leg in a state a state of flexure, as represented in his plate; and if the bone be broken transversely (which I suppose rarely happens) its *length* may be preserved with tolerable success. But, after a short time, the patient finds lying compleatly upon the side, a very uneasy position; the bed also sinks behind him, and he by degrees lies in some measure upon his back; the limb at the same time, perhaps, appearing perfectly straight; and the surgeon either satisfied with its appearance, or at a loss how to place it better. The consequence of this change of position is, that the upper portion of the thigh bone turns with the body, and when the union is compleated, the patient finds the whole of the limb below the fracture turned outwards; he walks with difficulty, and when he sits, the leg connected with the fractured thigh, cannot be placed perpendicularly with the foot flat upon the ground like the other, but it takes the position, more or less, as if it were to be placed upon the other knee. If the bone be fractured obliquely (the more common case) there will of course be a tendency in the lower portion, however well set, to slide upon the upper; but when the patient inclines backwards, and the upper portion of the thigh-bone turns with the body, the *edge* of one part will be upon the fractured surface of the other, and thus the points of contact being very few, the shortening of the limb will be almost inevitable. If the patient be laid upon his back, with the leg and thigh strait, according to the old method, (and the present practice indeed in fractures of the neck of the bone) the shortening of the limb is likely to be still greater; though the surgeon may in this way, prevent the deviation from the natural position of the foot.

Some *more certain* method, then, appears to be still highly desirable, of preventing the shortening and twisting of the limb, a *less disturbing* method of using the bed pan, and (in case the accident happens at a distance from a comfortable habitation) an *easy* method of moving the patient to some distance without danger.

I had often thought on this subject, and a variety of imaginary improvements occurred to me. At length I was called to a young man with *both* his thighs broken about five inches from the knee-joints. My first plan did not please me; but on the tenth day I placed him upon a machine which answered my expectations. At the end of the fifth week from the accident he was released from his confinement, with the bones perfectly united, the feet in their proper position, and the limbs of equal length. He suffered suffered no pain of any kind after he was placed in this machine, and is entirely free from lameness. It is right to observe, that the *use* of the machine in this case led to several very material improvements in its powers; for as it was constructed in haste, partly from preconceived notions, and principally for the use of the individual already mentioned, it was consequently in several respects defective and unaccommodating. I by no means offer it to the profession as a perfect machine, for I think it highly probable that farther improvements in practice might occur even to myself; but I offer it as affording the means of approximation to a good method of managing cases hitherto difficult, and too frequently unfortunate. It strikes me, that it would be worth trial in the melancholy cases of fracture of the lumbar vertebræ. In those cases there has hitherto appeared to me no chance of an union of the fractured portions of bone, from the sufferer's being necessarily moved so often in consequence of the palsied state of the sphincter ani and sphincter vesicæ. On this machine every attention of that sort might be paid to the patient, without his being disturbed; and the lower limbs being so well supported, by lessening the pressure upon the lower part of the spine, the bad effects of an irritating moisture and pressure combined, namely gangrene and its consequences, might I think be obviated.

It is not very easy to describe intelligibly any kind of complicated machinery. The description may seem sufficiently clear to the describer in consequence of his perfect knowledge of the thing which he describes, and yet it may be in reality so defective, as not to convey ideas sufficiently distinct to others. I am anxious to be understood, and with the help of figures will perform the task as well as I am able. If, however, my delineations (which are far from skilfully executed) together with one done by a friend in perspective, (rather less simple than the one which I have delineated) should not be thought to answer the purpose; as, I shall very shortly send a complete apparatus to the London Hospital to my friend Sir William Blizard, (who will I am sure, with his accustomed liberality, permit professional gentlemen and surgeons' instrument makers to see it) if it should be thought to deserve so much trouble, an artist may there make a more distinct drawing.

(AA. Fig. 1,) is a frame, a kind of bedstead, six feet in length, twenty inches wide and three inches thick. Three feet three inches of the upper part have laths across in the manner manner of a lath bedstead; on which is placed a hair mattrass (I) twenty inches wide, two feet nine inches long, and two inches and a half thick. This mattrass is secured or fixed to the frame by tapes, which are sewed far enough from the edge of the mattrass to admit of blankets, &c. being tucked under it. At the lower end of this mattrass is another at (K) fifteen inches wide and only six inches long, of the same thickness. This small mattrass is placed over a sort of trap door fastened by two small bolts, which cannot be seen in the figure, and under this trap door is a shelf fastened at one end by the button (F) and at the other by a hinge. When the bed-pan is to be used, the shelf is first unbuttoned, and hangs by its hinges; the trap door is then unbolted, and hangs the other way; the mattrass (K) sinks with it and is removed, the shelf is then buttoned up for the reception of the bed-pan, and the mattrass, trap door, &c. are afterwards replaced with little or no inconvenience to the patient. On each side, exactly where the mattrasses end, fixed to the middle of the lever (M) by a hinge, an oak socket (L) six inches and a half long, rises to form one side of a triangle; it is two inches square, and four inches of the lower part are bevilled off to admit of its depression towards the foot of the frame. This socket and the one on the opposite side are united by a cross frame, on which the lower parts of the fracture boxes rest. Each of these sockets has a longitudinal perforation, five-eighths of an inch in diameter, to admit a male screw (N) of the same diameter, and (with its double joint) thirteen inches and a half long, which passes thro' it and through a long opening in the lever (M) and a similar one in the main frame below, long enough to allow of the motion of the screw when the socket (L) is elevated or depressed. Another screw also passes through the main frame, to raise or lower the lever at (M), the use of which will be afterwards explained. On the top of each socket is a brass plate, one-eighth of an inch thick round the centre, which is perforated. On this plate a hollow steel screw (O) moves, for the purpose of turning up or down the male screw (N), and when it turns too stiffly for the fingers, the key (Fig. 5) may be used. The upper part of each male screw (N) terminates in a double joint, to allow of lateral as well as longitudinal motion, and this in a piece of iron (AA. Fig. 2) on which the upper part of the frame (P. Fig. 1) rests, (see also a compleat delineation of the under part of this frame Fig. 2), which frame forms the side of the triangular elevation on which the legs are placed.

placed. This piece of iron is so bent, that the centre of motion of the upper joint of the male screw (N) and the centre of motion of the joint of the fracture box at the knee (A. Fig. 3) are exactly the same, and is designed to prevent any alteration of the length between the knee and hip-joints when the base of the triangle is lengthened or shortened; a thing highly necessary to prevent anchylosis when the fracture is at the neck of the bone. The upper part of this frame (AA. Fig. 2) is reduced, to admit it between the socket, when the screws are let completely down, to reduce the triangle for the smallest sized fracture boxes. To each of the lower corners of this frame (P Fig. 1, and BB. Fig. 2) is fastened a bolt (Q) with a spring behind it, with notches to regulate its projection, and the extremity of the bolt made to fit the notches or rack work (R) on the main frame. This rack work is about fifteen inches long, the bolts are eight inches long. Thus a sort of triangular elevation is formed, the sides of which may be increased or diminished at pleasure. To prevent the bolts from slipping up and becoming shorter by any accident, a small screw on the top of the frame (P. Fig. 1) is visible, just below (P) and immediately over the upper part of the bolt. Upon this triangular or wedge-shaped elevation is placed a pair of leg and thigh fracture boxes, of a suitable size (Fig. 3 large size) near to, or as distant from each other by the use of thumb screws (4 Fig. 1 and Fig. 3) through the opening (C. Fig. 2) as the surgeon may think proper. The patient is laid upon his back, and when his legs and thighs are in the boxes, by means of the elevating screws (N and O. Fig. 1) in the socket (L) the side of the triangle parallel with the thighs may be with the greatest ease regulated, so as to suit the length of the limb, or to produce any degree of extension the surgeon may think necessary, in the most gentle and accurate manner; the *equal* length of the screws (N) being ascertained by means of a graduated measure. Before the involuntary action of the muscles ceases, the principal part of the *extending* force will be upon the upper portion of the gastrocnemius muscle. If this muscle be large, the heel will be considerably elevated from the right line. To support the heel and the lower part of the leg, the elevating screw (U. Fig. 1 and Fig. 3) is used to raise the lower part of the fracture box from (V) to (W) as much as may be needful; but when this elevation is considerable, as the extremities of the splints (which form the sides of the fracture boxes) will be relatively depressed, and their upper straps apt to press press upon the knee and instep, the buckles (1, 1, T. Fig. 1) must be loosened sufficiently to remedy this inconvenience, and the restraining button (2) will then prevent the too great separation of the splints at their lower edge. It will be necessary for the accommodation of different sized limbs, from the age of six or seven years to the largest adult size, to have three sets of fracture boxes. The part which *supports* the leg and thigh, viz. the bottom of the fracture box, is made of deal, about one inch thick, with what the carpenters call a rule joint (A. Fig. 3) at the bend of the knee, and turned without the usual angular projection. This supporting part of the two limbs is hollowed out a little, and the inequalities of the joint (occasioned by the hollowing out) rounded off. There is also a sliding footboard (X. Fig. 1, 3, 4, and 6) made to slide over [illegible] bottom cushion; and as the hollow for the heel is abou four inches long, it will accommodate legs of different lengths with the same cushion. There are also a pair of leg and a pair of thigh splints, forming the sides of the fracture boxes, (S and T. Fig. 1) made of thin American deal (which is the best, because most free from knots) half split through its substance in several places on the outside, with leather straps and buckles sewed to them. The buckles are all upon the outer splints, and the lower straps of the inner splints pass under the supporters (b. b. b. Fig. 3) and behind pieces of stout wire, like flat staples, as there represented. The inner thigh splint of the large size is of a proportionable width, but only ten inches and a half long; the outer (S. Fig. 1) fifteen inches and a half by nine, and six inches wide. The leg splints, of the large size also, (T. Fig. 1) are twenty-one inches at the top, and eighteen at the bottom in length, and five and a half wide. The supporter of the thigh (D. Fig. 3 and Fig. 6) is thirteen inches and a half long and five and a half wide in the widest part. The inner and lower part of this supporter is cut out, in the form of a quarter of a circle, as at (D. Fig. 6) for the convenience of the patient, and the edge thinned and smoothed off. The supporter of the leg from A to W (Fig. 3 and 6) when the lower part is not elevated, is nineteen inches long, and (except the narrowed part, where the foot board slides up and down) four and a half wide in the narrowest part. So that the sides of the supporters, when not bent at the joint (A. Fig. 3 and 6) form two right lines gradually approximating from D to W. The splints and the supporters, properly lined with wool, compresses, or cushions tacked to them, form very very easy beds for the limbs, and by means of the straps and buckles, secure them in the shortest time imaginable. The machine stands upon six legs, which in the figure done in perspective are made to take off occasionally; and the lower ends being put into staples, as there delineated, were designed to be used as handles, to move a patient some distance when necessary. It was also made to double for occasional package, which at the same time admitted of the elevation of the patient's shoulders, which is better done at B (Fig. 1). About three inches seems a pleasant elevation; a greater might perhaps occasion too much pressure upon the lower part of the spine. In this machine if both legs as well as both thighs were broken, they might I think be set with a fair chance of success, so far as the fractures merely are concerned; and by unbuckling two buckles only, the surgeon may dress a wound of any limb. I now come to the explanation of the use of the lever M (Fig. 1.) As it is of great consequence in fractures of or near the neck of the thigh-bone, that occasional flexion and extension of the knee and hip-joints should be made to prevent anchylosis; so is it of consequence that this should be done with as little motion of any other kind as possible. To prevent this injurious motion, it is necessary that the hinges of the sockets (L. Fig. 1) should be exactly level with the lower part of the patient; but as any kind of bedding, even a hair mattrass, must be more or less compressible, and that in proportion to the weight of the patient; it is also necessary that there should be some means of elevating and depressing the *hinge* part of the sockets, according to circumstances. The diagram (Fig. 7) will perhaps explain this necessity most clearly. The space between the parallel lines E D and C B may be considered as the mattrass. The line A B the socket and screw fixed to the main frame (without the intervention of a lever) at B; and the line AD the under part of the patient's thigh. Now if the limb be depressed to F, the sides of the angle EDA being less than the sides of the angle CBA, it will, on depression through the arc AF, approach to a right line more rapidly than the other; and unless the patient slides down after the fracture box, the lower part of his body will be raised from the mattrass from D to G, and the fracture box will be separated from the thigh from M to G.

Let AB and AD be two parallel lines, and let CB and ED be also two parallel lines. Let AF be the arc of the radius AB, and let B and F be united. Let IH be the arc of the radius ED, and let KL be the arc of the radius AD from from the point F; then let a line be drawn from E to the point where the circles cut each other at G, and let another line be drawn from G to F; then the sides of the angle EGF will be equal to the sides of the angle EDA, and the sides of the angle CBF will be equal to the sides of the angle CBA. Then the parallel lines AB and AD will form the triangle GMF, and the lines EG and ED will form the triangle EGD.

Military surgeons will be the best judges of the usefulness of this machine on service. There can be no doubt of its usefulness, unless I am greatly deceived, in military and other hospitals. On board ship, where the management of fractured thighs must, I imagine, be still more difficult than elsewhere, this machine, without any legs, might be fitted up in what is called a cot, with a part of the bottom and the sides of the cot to open occasionally. It might be made more than twenty inches wide, and more than six feet long, if thought necessary; for having as I hope, established the principles of its action, the detail of dimensions may be varied at pleasure. In all large ships, but more especially in his Majesty's service, such a machine must be very desirable, and would occupy no room worth mentioning, little more indeed than a hammock; and where it is not likely to be wanted for children, two sets of fracture boxes would be sufficient. The screws, &c. must of course, for this purpose, be made of brass, unless the Norwich composition of iron, &c. which is said not to rust, should be found strong enough to answer the purpose.

For hospital practice, two or three iron bedsteads, upon the same plan, might be fitted up, and (if the fracture boxes were kept ready lined) as soon as a patient's clothes were off, his limbs might be set completely in a few minutes.

It would be found not only useful, but (what is sometimes thought of greater consequence) *economical* for every parish work-house to be provided with such an apparatus, for if after a serious accident a man remain lame for life, he will most likely continue to be chargeable as long as he lives. Such a misfortune might sometimes be prevented by this means, and the parish relieved from the burthen of maintaining a disabled pauper.

Most surgeons have probably experienced the trouble occasioned by the splints being frequently displaced in fractures of the thigh; in this contrivance they will find no trouble of that sort.

I have

I have not mentioned the two projecting parts CC (Fig. 6), they are designed to prevent the edges of the splints sinking below the edge of the support. Where the thigh is so muscular as to rise above the splint, by means of the two lower buckles S (Fig. 1) the splints S may be raised sufficiently. As the inner thigh splint is too short to be opposite the lower strap of the outer, a strap goes across from one outside splint to the other. In Fig. 6, at b. b. b. are the places where the long straps of the inside splints pass under the supporters; at D a short strap is fastened to a button or pin, and the upper straps of the splints S (Fig. 1) are fastened in the same manner above A (Fig. 6), it being unnecessary for them to pass under the support.

I shall make no apology for the want of elegance in this description, my principal aim has been at perspicuity, which, on this occasion, I think of much more importance.

*Cheshunt, Herts.* I am, &c.

*Oct.* 25, 1805. E. HARROLD.

